# Supplementary material for: Pleiotropy of polygenic factors associated with focal and generalized epilepsy in the general population
Source: PLoS One. 2020 Apr 28;15(4):e0232292. doi: 10.1371/journal.pone.0232292 (PMC7188256; doi:10.1371/journal.pone.0232292)
Supplement: S2 Fig — Plotted are the PRS-phenome association results for GE and FE for all binary diseases of the nervous system in the UKB. The betas for GE-PRS are highlighted in blue, and for FE-PRS in red. P-values were calculated using a logistic regression model, adjusted for sex and the first four principal components of ancestry. The threshold to reject the null hypothesis was set to α = 5.95x10^-4^ after Bonferroni correction for 84 tests. Legend: UKB: UK Biobank, SE: standard error. (DOCX) [file pone.0232292.s002.docx]

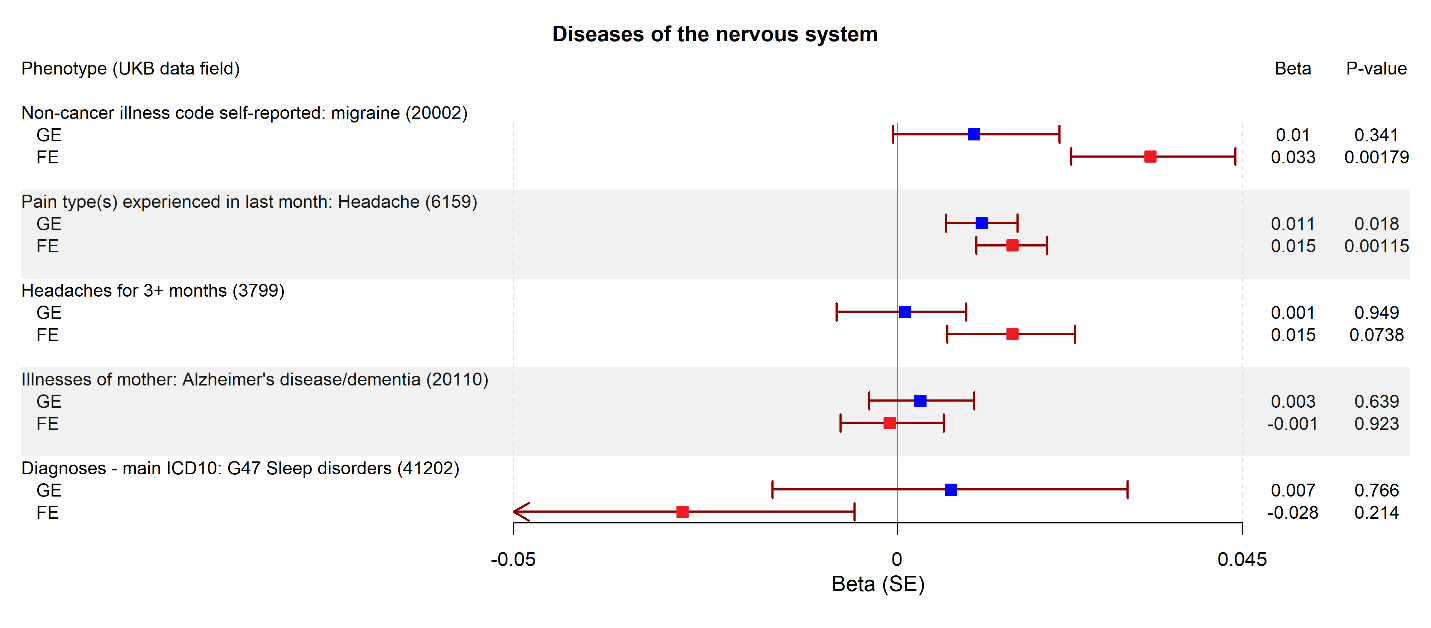


**Fig S2: Association between genetic risk for GE and FE and other diseases of the nervous system**

Plotted are the PRS-phenome association results for GE and FE for all binary diseases of the nervous system in the UKB. The betas for GE-PRS are highlighted in blue, and for FE-PRS in red. *P*-values were calculated using a logistic regression model, adjusted for sex and the first four principal components of ancestry. The threshold to reject the null hypothesis was set to α=5.95x10^-4^ after Bonferroni correction for 84 tests. Legend: UKB: UK Biobank, SE: standard error.
