# Supplementary material for: Pleiotropy of polygenic factors associated with focal and generalized epilepsy in the general population
Source: PLoS One. 2020 Apr 28;15(4):e0232292. doi: 10.1371/journal.pone.0232292 (PMC7188256; doi:10.1371/journal.pone.0232292)
Supplement: Attachment — Submitted filename: PLOS_ONE_Review_1-20.docx [file pone.0232292.s004.docx]

The authors present interesting analyses that demonstrate pleiotropic effects at the genome-wide level between PRS for generalized and focal epilepsy and a number of behavioral/cognitive traits. These analyses are valuable because they further our understanding of how genetic risk for epilepsy may play a role in other brain or cognitive phenotypes. In general, the article is well written, and analyses are performed to a high technical standard. However, I have a few major and minor comments that I would like addressed before I can recommend the article for publication.

**Comments:**

1. Can the authors distinguish their work more from the Brainstorm Consortium paper in *Science*? This paper also looks at genetic correlations between focal and generalized epilepsy and a number of phenotypes. Some of the results overlap with this paper (e.g., educational attainment), and some do not (e.g., neuroticism). The authors are using different methods to examine pleiotropy (PRS vs. LDSC regression), but it seems like results between the papers should overlap more. Along these lines, what are the advantages of using PRS over LDSC regression to examine pleiotropic effects?
2. Can the authors provide evidence that their results are robust to other clumping/pruning parameters or to the use of PRS created using LD Pred? There is no standard in the field for best methods to use when creating PRSs so I think some robustness checks along these lines are needed to show that the results are not sensitive to how the PRS were constructed.
3. Why do the authors only include 4 PCs in their analysis? Are the results robust to the inclusion of more PCs (10 seems to be more common in the literature)?
4. Why do the authors control for sex but not age?
5. In the figures that display the results, it would be helpful for the reader if the results that pass Bonferroni correction are noted in the figure somehow.
6. Can the authors test whether their findings are due to biological or mediated pleiotropy by controlling for whether or not an individual has seizures or has been diagnosed with epilepsy? For example, if correlations between the PRS and educational attainment are not significant after controlling for the epilepsy phenotype, this suggests mediated pleiotropy (e.g., see Schmitz et al., 2019). I think this is useful for clarifying whether or not the genetic risk is independent or working through the phenotype. Particularly in the case of educational attainment, an individual may not have completed much schooling because they struggle with seizures, rather than because genetic variants are independently affecting both phenotypes. Same for neuroticism--if an individual has epilepsy, they may be worried about have seizures in dangerous or inappropriate places and isolate themselves more as a result.
7. The PRS analyses are interesting, but they leave me wondering what genes may be driving these associations. Can the authors test for pleiotropic effects at the gene level for a few key epilepsy genes that have been implicated in the literature? In particular, the PRS results do not tell us much about the underlying biology behind the associations that may be useful from a clinical standpoint. In addition, even within ancestry, the weights from GWAS used to construct the PRS are likely biased by population stratification, assortative mating, or differences in environmental variance between groups (e.g., see Mostafavi et al., 2020). Because of these limitations, analyses at the gene region level would substantially enhance the findings reported in this paper.

**Cites:**

Mostafavi et al., 2020. Variable prediction accuracy of polygenic scores within an ancestry group *eLife*; 9:e48376.

Schmitz et al., 2019. Examining sex differences in pleiotropic effects for depression and smoking using polygenic and gene-region aggregation techniques. *American Journal of Medical Genetics Part B: Neuropsychiatric Genetics*, *180B*: 448-468.
