# Supplementary material for: Pleiotropy of polygenic factors associated with focal and generalized epilepsy in the general population
Source: PLoS One. 2020 Apr 28;15(4):e0232292. doi: 10.1371/journal.pone.0232292 (PMC7188256; doi:10.1371/journal.pone.0232292)
Supplement: Attachment — Submitted filename: EPI-PRS-UKB_Response_to_Reviewers_March19.docx [file pone.0232292.s005.docx]

We thank the referees for their comments, which we have addressed as follows:

5. Review Comments to the Author

*Please use the space provided to explain your answers to the questions above. You may also include additional comments for the author, including concerns about dual publication, research ethics, or publication ethics. (Please upload your review as an attachment if it exceeds 20,000 characters)*

**Reviewer #1**: This is the report of a wide retrospective study of pleiotropy of poligenic factors associated with focal and generalized epilepsy. The authors used data from an existing biobank including 334,310 individuals of European ancestry and found high polygenic risk scores for both epilepsies, with generalized epilepsies showing the largest effect sizes for personality traits associated with neuroticism and smoking and focal epilepsies inversely associated with education. Genetic factors associated with the two epilepsies showed similar patterns of correlation with the genetics of cortical morphology in a subsample (16,612 individuals).

The study findings are interesting and add to the existing knowledge on the complexity of the association between genetic and environmental factors in epilepsy. I have, however, a few major queries for the authors to address to provide convincing evidence on these purported associations. More specifically:

1. There is paucity of details on the individuals included in the biobank; the purposes of the biobank are not illustrated and there are no data on the way the information on each individual was collected; the Methods should be expanded to include these details;

**Response:** We thank the reviewer for this suggestion. We have added a description of the UK Biobank as given in the original UKB publications to the Methods section.

*Page 6, Methods, line 104, we added a new subchapter:*

*UK Biobank resource*

*The UKB is an open-access population-based prospective study with over 500,000 participants aged 40–69 years who were recruited in 2006–2010 [13]. Phenotypic data for >2000 traits & disorders were collected from questionnaires, physical measures, sample assays, accelerometry, multimodal imaging (e.g., MRI), and health records. All individuals of the UKB are genotyped with the Axiom array [12]. Of note, despite efforts to ensure a broad distribution across all health outcomes, there is evidence of enrichment of the UKB with healthy volunteers [14]. The healthy volunteer selection bias is likely to be particularly strong for mental disorders, where disorder status or symptoms may influence participation in research [15]. For this study, we used all individuals of European ancestry of the UKB that had genotypic data (N=334,310).*

2. The diagnosis of focal vs generalized epilepsy has been made according to the 2017 ILAE Classification; however, there is no indication of the criteria used and applied; the validity of this information must be also illustrated;

**Response:** We apologize for the lack of clarity in our description of the generation of PRS for focal vs generalized epilepsy in each of the selected UKB individuals. In addition to the UK Biobank resource description (subchapter “UK Biobank resource”, see above), we have rephrased the relevant part of the “PRS-Phenome association” method to emphasize that ILAE epilepsy classifications were part of the GWAS that delivered the SNP effect sizes needed for PRS calculations (ILAE et al., 2018).

*Page 7, Method “*PRS-Phenome association”, *paragraph 2, line 124 was changed from: “We then generated polygenic risk scores for generalized (GE-PRS) and focal epilepsy (FE-PRS) using SNP weights derived from summary statistics of the ILAE Consortium on Complex Epilepsies GWAS [17].” to “We then generated polygenic risk scores for generalized (GE-PRS) and focal epilepsy (FE-PRS) for all UKB individuals using single-nucleotide polymorphism (SNP) weights derived from summary statistics of the International League Against Epilepsy (ILAE) Consortium on Complex Epilepsies GWAS for GE and FE [17].”.*

3. As specified by the authors, “… individuals with a diagnosed brain disorder were excluded.” These exclusion criteria should be specified to put focal epilepsy (largely attributable to structural CNS injuries) in a more correct perspective.

**Response:** We apologize: The selection process took already place in the UKB project, which was targeting predominantly healthy participants for MRI screening in the first place (Miller et al., 2016 – PMID: 27643430). We have revised our wording to avoid misunderstanding that individuals with MRI data were explicitly screened in this study to exclude severe phenotypes.

*Page 8, Method “*Vertex-wise genetic correlation”, *line 147 was changed from: “Cortical reconstruction was performed in a UKB [12] subset of 16,612 individuals of European ancestry with T1-weighted MRI data, using Freesurfer [27]. Individuals with a diagnosed brain disorder were excluded.” to “Cortical reconstruction was performed in a UKB [12] subset of 16,612 predominantly healthy [26] individuals of European ancestry with T1-weighted MRI data, using Freesurfer [27]. Of note, the impact of confounding factors such as comorbid disorders or medication on cortical morphology was reduced by the MRI data acquisition from predominantly healthy participants in the UKB [25].”.*

Minor points:

1. Introduction, lines 75 and 76: The sentence in question should be accompanied by a reference;

**Response:** We thank the reviewer for this suggestion. We added the corresponding reference.

*Page 5, Introduction paragraph 1, line 76: Fisher et al., 2014; PMID: 24730690; “ILAE official report: a practical clinical definition of epilepsy” was added as a reference.*

2. Methods, line 113: SNP and ILAE should be spelled out;

**Response:** We spelled out SNP and ILAE.

*Page 7, Method “*PRS-Phenome association”, *paragraph 2, line 124 was changed from: “We then generated polygenic risk scores for generalized (GE-PRS) and focal epilepsy (FE-PRS) using SNP weights derived from summary statistics of the ILAE Consortium on Complex Epilepsies GWAS [17].” to “We then generated polygenic risk scores for generalized (GE-PRS) and focal epilepsy (FE-PRS) for all UKB individuals using single-nucleotide polymorphism (SNP) weights derived from summary statistics of the International League Against Epilepsy (ILAE) Consortium on Complex Epilepsies GWAS for GE and FE [17].”.*

3. Methods, line 116: “a SNP” should perhaps read “an SNP”;

**Response:** We have changed our wording to avoid this uncertainty and improve the overall clarity.

*Page 7, Method “*PRS-Phenome association”, *paragraph 2, line 129 was changed from: “When a SNP was not present in the UKB genotype data, we attempted to identify a proxy …” to “When a GWAS SNP was not present in the UKB genotype data, we attempted to identify a proxy …”.*

4. Results, lines 145-147: The two sentences are a repetition with what said in the Methods and should be deleted;

**Response:** We agree and have removed the two leading sentences of the first results section.

*Page 9, Result “*Epilepsy-PRS are associated with brain or cognitive function-related traits”, *paragraph 1, line 166, we removed: “To identify brain or cognitive function-related traits that are associated with epilepsy, we conducted a phenome association study using GE- and FE-PRS. We tested both epilepsy PRS for association with 42 traits related to cognitive function in 334,310 individuals of the UKB.”.*

5. Results, line 215: “parameter” should read “parameters”;

**Response:** We have corrected our spelling accordingly.

*Page 12, Result “*GE and FE show similar patterns of genetic correlation at vertex-level with cortical thickness and cortical surface area”, *line 238 was changed from: “The maps for GE were weakly correlated with the maps for FE for both parameter: cortical thickness … and cortical surface area …” to “The maps for GE were weakly correlated with the maps for FE for both parameters: cortical thickness … and cortical surface area …”.*

6. References, lines 331 and 360: Is the journal’s name correct?

**Response:** We thank the reviewer for spotting the formatting difference of the journal names.

*Page 16, reference 2, line 328 was changed from: “The Lancet Neurology” to “Lancet Neurol”.*

*Page 18, reference 17, line 373 was changed from: “Nature Communications” to “Nat Commun”.*

**Reviewer #2**: The authors present interesting analyses that demonstrate pleiotropic effects at the genome-wide level between PRS for generalized and focal epilepsy and a number of behavioral/cognitive traits. These analyses are valuable because they further our understanding of how genetic risk for epilepsy may play a role in other brain or cognitive phenotypes. In general, the article is well written, and analyses are performed to a high technical standard. However, I have a few major and minor comments that I would like addressed before I can recommend the article for publication.

Comments (in no particular order):

1. Can the authors distinguish their work more from the Brainstorm Consortium paper in Science? This paper also looks at genetic correlations between focal and generalized epilepsy and a number of phenotypes. Some of the results overlap with this paper (e.g., educational attainment), and some do not (e.g., neuroticism). The authors are using different methods to examine pleiotropy (PRS vs. LDSC regression), but it seems like results between the papers should overlap more. Along these lines, what are the advantages of using PRS over LDSC regression to examine pleiotropic effects?

**Response:** We agree with the reviewer that our results were not discussed in relation to the Brainstorm Consortium paper. We added a corresponding section to our discussion, which is discussing the topic raised by the reviewer.

*Page 13, Discussion paragraph 1, line 254, we added: “Our results confirm the genetic correlation between intelligence (as a proxy for educational attainment) and epilepsy, which is the only genetic correlation of epilepsy shown as significant in several studies [3,17]. The genetic pleiotropy between epilepsy and neuroticism observed in this study was missed in a larger study that employed linkage disequilibrium (LD) score regression [3], showing the value of examining pleiotropy with different methods. Currently, there is no single method that performs best for all possible trait pairs and study parameters [30]”.*

2. Can the authors provide evidence that their results are robust to other clumping/pruning parameters or to the use of PRS created using LD Pred? There is no standard in the field for best methods to use when creating PRSs so I think some robustness checks along these lines are needed to show that the results are not sensitive to how the PRS were constructed.

**Response:** We agree that there is no established best practice for the generation of PRS. We have added a section to the discussion that provides support for the clumping/pruning procedure over LDpred in epilepsy.

*Page 14, Discussion paragraph 2, line 274, we added: “The use and generation of PRS is a rapidly developing field, with no established best-practice method. We selected the LD-clumping and P-value thresholding method to generate PRS based on a known excellent performance in neurological and psychiatric disorders [35–37]. A commonly used alternative is LDpred, a method that accounts for LD between SNPs and thus allows joint modeling with the potential of improvements in the prediction power [38]. However, the most significant improvements by joint modeling tend to be in diseases with multiple variants in LD that have independent effects (e.g., multiple sclerosis, rheumatoid arthritis, type I diabetes with associated HLA variants) [39]. In epilepsy, variants with independent effects within one LD region have not been demonstrated [17].”.*

3. Why do the authors only include 4 PCs in their analysis? Are the results robust to the inclusion of more PCs (10 seems to be more common in the literature)?

**Response:** We have added an explanation for using only four PCs in our “PRS-Phenome association” method. Price et al. (2006) originally suggested the use of 10 PCs for GWASs. However, more recently, the usage of four PCs emerged as a common praxis for PRS association statistics since the milestone publications of Khera and Kathiresan in Nature Genetics (PMID: 30104762) and the New England Journal of Medicine (PMID: 27959714).

*Page 8, Method “*PRS-Phenome association”, *paragraph 3, line 140, we added: “We selected only four PCs, following the example in Khera et al. (2018) [22], and to avoid overfitting when too many PCs are included association model [23,24].”.*

4. Why do the authors control for sex but not age?

**Response:** For consistency, we applied the same covariates as in the logistic regression model from our previous study (Leu et al., 2019 – PMID: 31608925) that successfully demonstrated the role of PRS in epilepsy.

5. In the figures that display the results, it would be helpful for the reader if the results that pass Bonferroni correction are noted in the figure somehow.

**Response:** We thank the reviewer for this suggestion. We made corresponding adjustments to all figures. *P*-values that remain significant after Bonferroni correction are now highlighted with an asterisk.

**Fig 1. Association between genetic risk for GE and FE and educational attainment phenotypes.**

**Fig 2. Association between genetic risk for GE and FE and neuroticism-related traits.**

**Fig 3. Association between genetic risk for GE and FE and mental and behavioral disorders due to substance abuse.**

**Fig 4. Association between genetic risk for GE and FE and nonpsychotic mental disorders.**

6. Can the authors test whether their findings are due to biological or mediated pleiotropy by controlling for whether or not an individual has seizures or has been diagnosed with epilepsy? For example, if correlations between the PRS and educational attainment are not significant after controlling for the epilepsy phenotype, this suggests mediated pleiotropy (e.g., see Schmitz et al., 2019). I think this is useful for clarifying whether or not the genetic risk is independent or working through the phenotype. Particularly in the case of educational attainment, an individual may not have completed much schooling because they struggle with seizures, rather than because genetic variants are independently affecting both phenotypes. Same for neuroticism--if an individual has epilepsy, they may be worried about have seizures in dangerous or inappropriate places and isolate themselves more as a result.

**Response:** We apologize for the lack of clarity in our original description of the UKB resource and the selection of UKB individuals. In response to the points 1.) and 3.) of Reviewer #1, we have added details on the individuals included in the UKB. These details answer the point raised by the reviewer. In short: a healthy volunteer selection bias in the UKB has been previously reported (Fry et al., 2017 – PMID: 28641372). This selection bias is likely to be particularly strong for mental disorders (Knudsen et al., 2010 – PMID: 20843863). Therefore, it is unlikely to find individuals with severe epilepsy phenotypes among UKB participants.

*Page 6, Methods, line 104, we added a new subchapter:*

*UK Biobank resource*

*The UKB is an open-access population-based prospective study with over 500,000 participants aged 40–69 years who were recruited in 2006–2010 [13]. Phenotypic data for >2000 traits & disorders were collected from questionnaires, physical measures, sample assays, accelerometry, multimodal imaging (e.g., MRI), and health records. All individuals of the UKB are genotyped with the Axiom array [12]. Of note, despite efforts to ensure a broad distribution across all health outcomes, there is evidence of enrichment of the UKB with healthy volunteers [14]. The healthy volunteer selection bias is likely to be particularly strong for mental disorders, where disorder status or symptoms may influence participation in research [15]. For this study, we used all individuals of European ancestry of the UKB that had genotypic data (N=334,310).*

*Page 8, Method “*Vertex-wise genetic correlation”, *line 147 was changed from: “Cortical reconstruction was performed in a UKB [12] subset of 16,612 individuals of European ancestry with T1-weighted MRI data, using Freesurfer [27]. Individuals with a diagnosed brain disorder were excluded.” to “Cortical reconstruction was performed in a UKB [12] subset of 16,612 predominantly healthy [26] individuals of European ancestry with T1-weighted MRI data, using Freesurfer [27]. Of note, the impact of confounding factors such as comorbid disorders or medication on cortical morphology was reduced by the MRI data acquisition from predominantly healthy participants in the UKB [25].”.*

7. The PRS analyses are interesting, but they leave me wondering what genes may be driving these associations. Can the authors test for pleiotropic effects at the gene level for a few key epilepsy genes that have been implicated in the literature? In particular, the PRS results do not tell us much about the underlying biology behind the associations that may be useful from a clinical standpoint. In addition, even within ancestry, the weights from GWAS used to construct the PRS are likely biased by population stratification, assortative mating, or differences in environmental variance between groups (e.g., see Mostafavi et al., 2020). Because of these limitations, analyses at the gene region level would substantially enhance the findings reported in this paper.

**Response:** We agree with the reviewer that identifying key genes that mediate most of the observed pleiotropy would be of high interest. As mentioned by the reviewer, such genes could be known epilepsy genes, or, as implied by the phenomenon of pleiotropy, genes associated with neurological or psychiatric traits. However, identifying the key players out of the large variety of potential pleiotropic genes would imply extensive testing (with the corresponding correction for multiples testing) and is out of the scope of this study. The main scope of our study was to broaden the (still elusive) knowledge basis of pleiotropic effects between epilepsy and other brain disorders and to explore the impact of genetic risk for epilepsy in predominantly healthy individuals. Finally, cross-disorder common or rare variant association analyses will be better powered to identify pleiotropic genes than PRS-based studies.

*Cites:*

*Mostafavi et al., 2020. Variable prediction accuracy of polygenic scores within an ancestry group eLife; 9:e48376.*

*Schmitz et al., 2019. Examining sex differences in pleiotropic effects for depression and smoking using polygenic and gene-region aggregation techniques. American Journal of Medical Genetics Part B: Neuropsychiatric Genetics, 180B: 448-468.*
